# Association of Resistance With Frailty Among Components of the FRAIL Scale in Geriatric Trauma Patients

**DOI:** 10.7759/cureus.107947

**Published:** 2026-04-29

**Authors:** Beverly L Hersh, Katie Lee, Katie Frank, Taylor Moglia, Peter Erickson, Brian Frank

**Affiliations:** 1 Trauma and Acute Care Surgery, Geisinger Commonwealth School of Medicine, Scranton, USA; 2 Statistics, Geisinger Health System, Danville, USA; 3 Trauma Surgery, Geisinger Community Medical Center, Scranton, USA

**Keywords:** frail questionnaire, frailty, geriatric trauma, risk stratification, stair climbing

## Abstract

Background

The FRAIL questionnaire is a validated screening tool for frailty in geriatric trauma patients that has been associated with functional outcomes and mortality. It includes five components: Fatigue, Resistance, Aerobic, Illness, and Loss of weight. This study aims to determine whether a single component was more strongly associated with the total frailty score than the other components in geriatric trauma patients.

Methods

A retrospective chart review was conducted on 613 geriatric trauma patients admitted between June 2023 and November 2024. Patient demographics, injury severity scores (ISS), and FRAIL questionnaire responses were obtained from electronic health records. Spearman’s rank correlation coefficient (ρ) was used to assess the relationship between each FRAIL component and total FRAIL score. A bootstrap 95% confidence interval (CI) was calculated using 10,000 replicates to evaluate statistical significance.

Results

All FRAIL components demonstrated positive associations with the total FRAIL score, with CIs that did not include zero. Corrected item-total correlation (CITC) analysis showed that resistance (ability to climb a flight of stairs) had the highest correlation with the remaining FRAIL components (ρ = 0.31, 95% CI: 0.24-0.39), followed by aerobic capacity (ρ = 0.27, 95% CI: 0.20-0.35), fatigue (ρ = 0.22, 95% CI: 0.15-0.30), and weight loss (ρ = 0.13, 95% CI: 0.06-0.20). Illness burden demonstrated no meaningful correlation with the other FRAIL components (ρ = 0.00, 95% CI: -0.08 to 0.08).

Conclusions

Resistance, specifically the ability to climb a flight of stairs, demonstrated the highest correlation with total FRAIL score among the five FRAIL components in this cohort of geriatric trauma patients. This finding suggests that resistance may reflect an important functional dimension of frailty, although further validation is needed to determine its clinical utility as a screening measure.

## Introduction

Frailty is increasingly recognized as a critical determinant of outcomes in geriatric trauma patients. Prior studies have demonstrated that frailty is a predictor of poor functional outcomes and mortality in this cohort [1,2]. Frailty has been shown to influence one-year functional status and mortality rates [1]. Additionally, frailty has been associated with increased rates of in-hospital complications, ICU admissions, and adverse discharge dispositions [3-4]. 

Recent evidence highlights the importance of targeted frailty interventions, as implementing frailty assessment programs in surgical patients has been associated with a reduction in postoperative complications and improved patient outcomes [5]. The 2023 World Society of Emergency Surgery (WSES) guidelines on geriatric trauma management, published in 2024, emphasize frailty as a major risk factor for mortality and adverse discharge outcomes [6]. 

The FRAIL questionnaire is a validated screening tool for frailty assessment in geriatric trauma patients and consists of five components: Fatigue, Resistance, Aerobic capacity, Illness, and Loss of weight. Each component is scored as present or absent, with a total score of ≥3 indicating frailty [1,2]. Prior studies have demonstrated that higher FRAIL scores are associated with clinically meaningful outcomes in older trauma populations. In a retrospective cohort of 2,862 older adults with traumatic injuries, patients classified as frail by the FRAIL index had increased adjusted odds of trauma team activation (OR 2.5, 95% CI 1.86-3.23), inpatient admission (OR 3.1, 95% CI 2.28-4.12), in-hospital death (OR 3.7, 95% CI 1.07-12.62), and discharge to rehabilitation (OR 2.2, 95% CI 1.71-2.91) [3]. 

Given its simplicity, the FRAIL questionnaire is a practical tool for bedside screening. However, while the FRAIL score has been shown to be associated with the outcomes noted above, it remains unclear whether one FRAIL component is more strongly associated with overall frailty burden than the others. Identifying a single component most closely associated with the total FRAIL score could help clarify which domains contribute most prominently to frailty in geriatric trauma patients and inform the development of more efficient screening approaches. This study aims to determine whether one component of the FRAIL questionnaire has a stronger association with the total FRAIL score than the other components. 

This article was previously posted to the Research Square preprint server on October 8, 2025.

## Materials and methods

Study design and population 

This was a retrospective cohort study, reviewed by the Institutional Review Board (IRB) of Geisinger Health System, Danville, PA, USA, and deemed exempt from ethical approval due to the use of de-identified, routinely collected data, and additionally approved with a waiver of informed consent due to the retrospective nature of the analysis. Ethical approval and consent were ruled as exempt by Geisinger IRB under number 2023-1545. We evaluated geriatric trauma patients admitted to Geisinger Community Medical Center, a Level II trauma center in Scranton, PA, USA, between June 2023 and November 2024. Eligible patients were aged 65 years and older at the time of admission. Data were extracted from the institution’s electronic health record system and trauma registry. Patients with repeat admissions within the study period were identified, and only the first (index) admission during this timeframe was included, resulting in a final cohort of 613 unique geriatric trauma patients. All available patients meeting criteria during the study period were included. 

Frailty assessment 

Frailty was assessed using the FRAIL questionnaire [1], a validated five-item screening tool consisting of the following binary components which are as follows: Fatigue: self-reported difficulty performing daily activities such as housework or climbing stairs; Resistance: inability to climb a flight of stairs; Aerobic capacity: inability to walk one block; Illness: presence of five or more chronic medical conditions, including hypertension, diabetes, coronary artery disease, congestive heart failure, chronic kidney disease, dementia, peripheral vascular disease, and active malignancy, identified using documented conditions in the electronic health record; Loss of weight: unintentional weight loss exceeding 5% of body weight within the past six months. 

Each component was assigned a score of 1 if present and 0 if absent, resulting in a total FRAIL score ranging from 0 to 5. Patients were classified as robust (score = 0), prefrail (score 1-2), or frail (score ≥3), in accordance with established FRAIL scale definitions [2]. 

Data collection 

Demographic information, comorbidity profiles, injury characteristics, and clinical outcomes were obtained from the electronic health record through protected data mining queries conducted by the Geisinger Surgical Institute, Scranton, PA, USA. These queries were performed by designated Health Data Analysts with IRB-approved funding support. Comorbidities included conditions such as hypertension, diabetes, coronary artery disease, congestive heart failure, chronic kidney disease, dementia, and active malignancy. Polypharmacy was defined as the use of five or more home medications, excluding over-the-counter medications. Mechanism of injury (MOI), ICU admission, hospital length of stay (LOS), and discharge disposition were also extracted from the electronic health record. Hospital LOS was defined as the number of days from admission to discharge. Discharge disposition was categorized as home, skilled nursing facility, rehabilitation facility, or other.

Statistical analysis 

Descriptive statistics were used to summarize patient characteristics, with medians and interquartile ranges (IQR) reported for continuous variables and counts with percentages for categorical variables. To assess the relationship between each individual FRAIL item and total FRAIL score, Spearman’s rank correlation coefficients (ρ) were calculated. Analyses were performed using available data for each variable, and patients with missing data were excluded from the corresponding analyses. Corrected item-total correlations (CITC) were used to evaluate the correlation between an individual item and the total score with that item excluded, providing an estimate of the item’s association with the remaining FRAIL components. A 95% confidence interval (CI) for each correlation coefficient was estimated using basic bootstrap resampling with 10,000 replicates, and correlations were considered statistically significant if the CI did not include zero. Statistical analyses were performed using R (version 4.3.1, The R Core Team, R Foundation for Statistical Computing, Vienna, Austria). Cls were derived using basic bootstrap resampling with 10,000 replicates, providing robust estimates of Spearman’s correlation coefficients for ordinal variables. No subgroup or interaction analyses were performed due to the descriptive scope of the study.

## Results

A total of 613 geriatric trauma patients aged 65 years and older were included in the final analysis. The median age was 80 years (IQR 74-87), and 60% (n = 370) of patients identified as female (Table 1). The majority sustained blunt injuries (92.5%, n = 567), with ground-level falls representing the most common mechanism (61%). Other mechanisms included falls down stairs (7.5%), motor vehicle collisions (6.5%), falls from height (2.3%), pedestrian strikes (2.0%), and other less frequent mechanisms. A total of 18.9% (n = 116) of patients were admitted to the ICU. 

**Table 1:** Demographics and health history of the study group (n = 613) ^1^Median (Q1, Q3); n (%); N/A: not available

Variable	
	(n = 613)^1^
Age (years)	80 (74, 87)
Gender	
Female	370 (60%)
Male	243 (40%)
BMI	26.2 (22.5, 30.2)
(# NA/Missing)	38
Obesity	152 (26%)
(# NA/Missing)	38
Smoking status	
Current	51 (9.2%)
Former	220 (40%)
Never	284 (51%)
(# NA/Missing)	58
Smokeless tobacco status	
Former	12 (2.3%)
Never	510 (98%)
(# NA/Missing)	91
Congestive heart failure	185 (32%)
(# NA/Missing)	37
Arrhythmias	240 (42%)
(# NA/Missing)	37
Myocardial infarction	92 (16%)
(# NA/Missing)	37
Heart disease	111 (19%)
(# NA/Missing)	37
Cardiac surgery in the prior six months	14 (2.3%)

Regarding comorbidities, 73.5% (n = 451) of patients had at least one chronic medical condition. The most prevalent were hypertension (58.1%, n = 356), diabetes mellitus (27.2%, n = 167), chronic kidney disease (18.4%, n = 113), and coronary artery disease (16.6%, n = 102). A smaller subset had a history of stroke or transient ischemic attack (10.3%, n = 63), dementia (14.0%, n = 86), or active cancer (6.7%, n = 41). Polypharmacy, defined as the use of five or more medications, was present in 61.2% (n = 375) of the cohort (Table 1).

Frailty was assessed using the FRAIL scale, consisting of five binary components: Fatigue, Resistance, Aerobic capacity, Illness burden, and Loss of weight. Among the cohort, 46% of patients endorsed fatigue, 61% were unable to climb a flight of stairs (resistance), 80% had difficulty walking one block (aerobic capacity), 28% had five or more chronic illnesses, and 4.7% reported unintentional weight loss. Based on cumulative FRAIL scores, 44% (n = 267) of patients were classified as frail (score ≥3), 47% (n = 291) as prefrail (score 1-2), and 9.0% (n = 55) as robust (score = 0) (Table 2). 

**Table 2:** FRAIL-related items (n = 613) ^1^n (%)

Variable	Overall
	(n = 613)^1^
F- Fatigue	281 (46%)
R- Resistance	374 (61%)
A- Aerobic	490 (80%)
I- Illness	173 (28%)
L- Loss of Weight	29 (4.7%)
FRAIL score	
0	55 (9.0%)
1	104 (17%)
2	187 (31%)
3	207 (34%)
4	52 (8.5%)
5	8 (1.3%)
FRAIL category	
Robust (0)	55 (9.0%)
Prefrail (1-2)	291 (47%)
Frail (3-5)	267 (44%)

**Figure 1:**
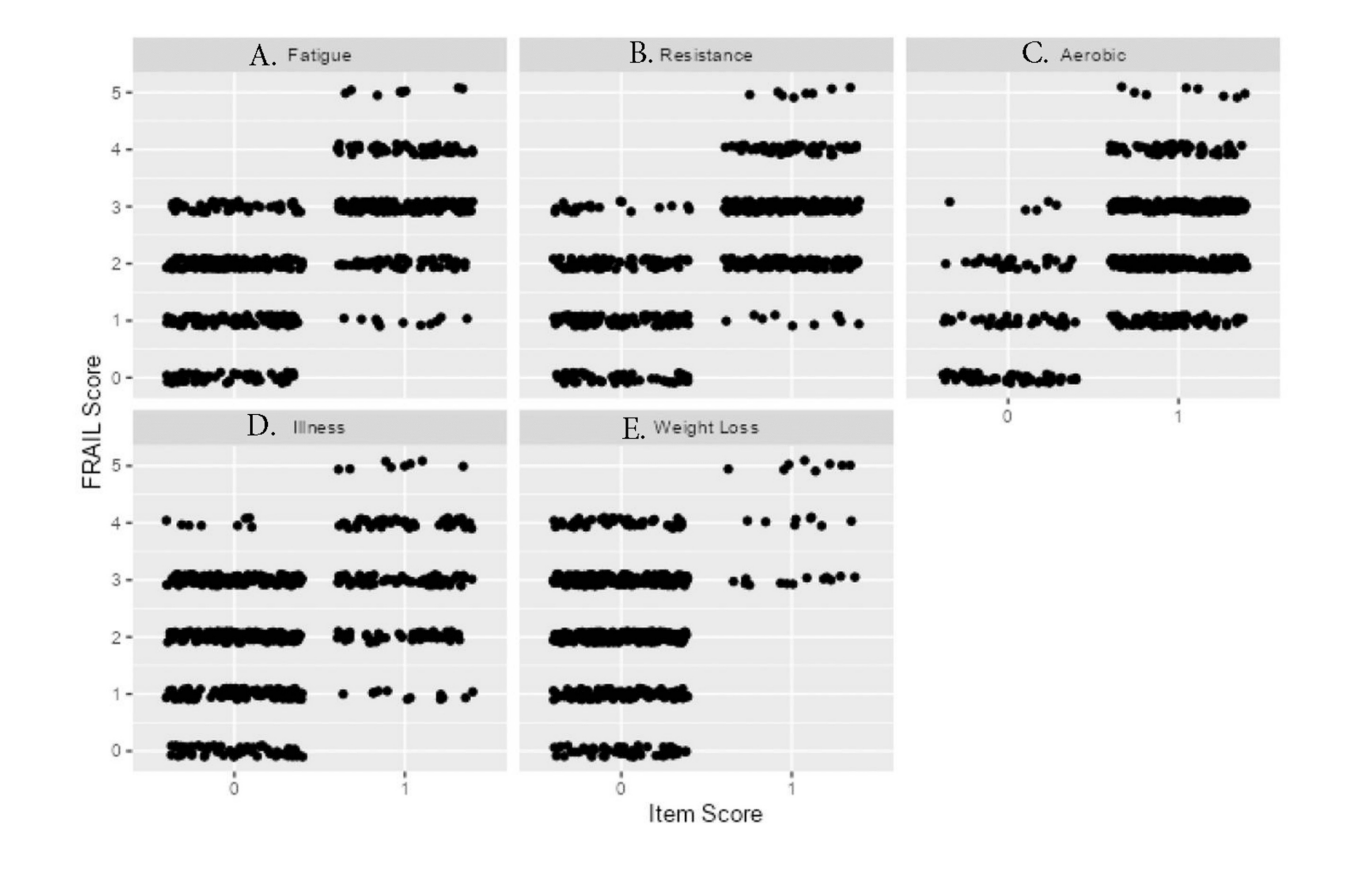
Jitter plots of FRAIL scale items and FRAIL score. Relationship between individual FRAIL scale components and total FRAIL score in geriatric trauma patients (n = 613). Each subplot displays jittered data points comparing binary component scores (0 = Absent, 1 = Present) across five FRAIL domains: (A) Fatigue, (B) Resistance, (C) Aerobic capacity, (D) Illness, and (E) Loss of Weight, to Cumulative FRAIL scores (0-5). Each point represents an individual patient, with horizontal jitter applied to reduce over-plotting. Among individual components, resistance shows the highest correlation.

CITCs were used to assess the association between each FRAIL component and the total score with that item removed. Resistance demonstrated the highest correlation (ρ = 0.31, 95% CI: 0.24-0.39), followed by aerobic capacity (ρ = 0.27, 95% CI: 0.2-0.35), fatigue (ρ = 0.22, 95% CI: 0.15-0.3), and weight loss (ρ = 0.13, 95% CI: 0.06-0.2). Illness burden showed no meaningful correlation with the remaining FRAIL components (ρ= 0.00, 95% CI: -0.08-0.08). Overall, these findings indicate modest associations between individual FRAIL items and the remaining components of the FRAIL scale. A jitter plot illustrating the correlation of each variable can be seen in Figure 1. 

Patients classified as frail had longer median hospital LOS compared to pre-frail and robust patients (6.05 (3.93-9.08) days vs. 4.75 (2.94-7.67) days vs 3.90 (1.21-5.08) days, p < 0.001). Similarly, discharge to skilled nursing facilities was more common among frail patients (58.3%) compared to pre-frail (41.8%) and robust patients (13.3%) (p < 0.001).

## Discussion

In this retrospective cohort study of geriatric trauma patients, we found that frailty was common, with 44% of patients classified as frail using the FRAIL scale. Among the five components of the FRAIL questionnaire, resistance (defined as the inability to climb a flight of stairs) demonstrated the highest CITC with the overall frailty score among the FRAIL components. The magnitude of this correlation was modest, explaining a limited proportion of variance in the overall FRAIL score. However, relative to the other FRAIL components, this finding suggests that lower extremity strength and functional mobility may represent an important dimension of frailty in this population. 

These results are consistent with prior literature identifying impaired physical function as a hallmark of frailty. Previous studies have shown that frailty is associated with poor outcomes in geriatric trauma patients, including higher rates of ICU admission, in-hospital complications, and adverse discharge dispositions [1-3]. Our study builds on this literature by identifying resistance as the FRAIL component most closely associated with other FRAIL components in this cohort. Whether resistance alone has sufficient predictive performance to function as a standalone screening measure would require prospective validation using appropriate performance metrics. Future analyses should examine whether resistance is independently associated with key clinical outcomes, such as ICU admission, LOS, or discharge disposition, using multivariable approaches. 

Notably, illness burden showed no correlation with the remaining FRAIL components, suggesting that multimorbidity may reflect a domain distinct from functional frailty in this population. While the other FRAIL components primarily capture aspects of physical function and physiologic reserve, the illness component reflects comorbidity burden, which may not directly correspond to functional impairment in all patients. This finding highlights the potential multidimensional nature of frailty and warrants further investigation into how individual FRAIL components contribute to risk stratification in geriatric trauma populations. 

Routine assessment of stair-climbing ability may offer useful descriptive information about functional status in older trauma patients. However, whether this single item can reliably identify high-risk patients or guide targeted interventions requires further study. Future work should evaluate whether this approach can facilitate earlier identification of high-risk patients and support implementation of targeted interventions such as physical therapy, early mobilization, or mobility-based discharge planning. This approach aligns with recent evidence highlighting the impact of structured frailty programs on postoperative outcomes, including reduced complication rates and improved discharge trajectories [4,5].

This study has several limitations. First, it is retrospective and based on data extracted from electronic health records, which introduces the potential for misclassification or documentation bias. Second, frailty was assessed via a self-reported questionnaire rather than objective performance-based measures. Several studies have attempted to validate the predictive accuracy of various frailty assessment tools in older trauma populations, though no single model has emerged as clearly superior across all outcomes. For example, a prospective comparison of the FRAIL Scale, Trauma-Specific Frailty Index (TSFI), and Clinical Frailty Scale (CFS) found that all were associated with increased fall risk, but no individual tool significantly outperformed the others in predictive performance [7,8]. While the FRAIL scale is validated and feasible for bedside use, further research is needed to compare its predictive performance against timed mobility tests or other objective metrics, specifically in trauma populations. Furthermore, frailty screening tools can be time-consuming or burdensome to apply in acute care settings, limiting their widespread use. Prior studies have highlighted the need for simpler, high-yield screening approaches in trauma care environments [9,10].

Additionally, the observed associations between frailty, LOS, and discharge to skilled nursing facilities should be interpreted with caution. Preinjury residence was not available in our dataset, and some patients discharged to a skilled nursing facility may have been admitted from such facilities at baseline. Furthermore, hospital length of stay may be influenced by discharging patient complexity, insurance authorization, and placement delays, rather than frailty alone. 

Despite these limitations, our findings highlight the value of resistance as an informative functional component within the FRAIL framework. Future prospective studies should investigate whether brief resistance-based assessments can provide incremental value alongside established frailty tools and whether they could be used to inform intervention strategies for older adults after injury.

## Conclusions

Resistance, defined as the inability to climb a flight of stairs, emerged as the FRAIL component which demonstrated the highest correlation with overall frailty in this cohort of geriatric trauma patients. This finding reinforces the centrality of lower extremity strength and mobility within the broader frailty construct. Future prospective studies are needed to determine whether resistance alone can provide incremental value alongside established frailty tools, and whether they are associated with clinically meaningful outcomes in geriatric trauma populations.
